# Evidence for alternative quaternary structure in a bacterial Type III secretion system chaperone

**DOI:** 10.1186/1472-6807-10-21

**Published:** 2010-07-15

**Authors:** Michael L Barta, Lingling Zhang, Wendy L Picking, Brian V Geisbrecht

**Affiliations:** 1 Division of Cell Biology and Biophysics, School of Biological Sciences, University of Missouri-Kansas City, Kansas City, MO, USA; 2 Department of Microbiology and Molecular Genetics Oklahoma State University, Stillwater, OK, USA

## Abstract

**Background:**

Type III secretion systems are a common virulence mechanism in many Gram-negative bacterial pathogens. These systems use a nanomachine resembling a molecular needle and syringe to provide an energized conduit for the translocation of effector proteins from the bacterial cytoplasm to the host cell cytoplasm for the benefit of the pathogen. Prior to translocation specialized chaperones maintain proper effector protein conformation. The class II chaperone, Invasion plasmid gene (Ipg) C, stabilizes two pore forming translocator proteins. IpgC exists as a functional dimer to facilitate the mutually exclusive binding of both translocators.

**Results:**

In this study, we present the 3.3 Å crystal structure of an amino-terminally truncated form (residues 10-155, denoted IpgC^10-155^) of the class II chaperone IpgC from *Shigella flexneri*. Our structure demonstrates an alternative quaternary arrangement to that previously described for a carboxy-terminally truncated variant of IpgC (IpgC^1-151^). Specifically, we observe a rotationally-symmetric "head-to- head" dimerization interface that is far more similar to that previously described for SycD from *Yersinia enterocolitica *than to IpgC^1-151^. The IpgC structure presented here displays major differences in the amino terminal region, where extended coil-like structures are seen, as opposed to the short, ordered alpha helices and asymmetric dimerization interface seen within IpgC^1-151^. Despite these differences, however, both modes of dimerization support chaperone activity, as judged by a copurification assay with a recombinant form of the translocator protein, IpaB.

**Conclusions:**

From primary to quaternary structure, these results presented here suggest that a symmetric dimerization interface is conserved across bacterial class II chaperones. In light of previous data which have described the structure and function of asymmetric dimerization, our results raise the possibility that class II chaperones may transition between asymmetric and symmetric dimers in response to changes in either biochemical modifications (e.g. proteolytic cleavage) or other biological cues. Such transitions may contribute to the broad range of protein-protein interactions and functions attributed to class II chaperones.

## Background

Type III secretion systems (TTSSs) use a conserved apparatus (TTSA) to provide an energy-driven conduit from a bacterium to the cell membrane and cytoplasm of targeted eukaryotic cells[1]. A hallmark of TTSSs is the presence of two secreted translocators that assume a position at the tip of the TTSA needle to form a pore in the host cell membrane[2]. Once the mature tip complex has formed, the conduit is completed and vectorial transfer of a pathogen-specific repertoire of secreted effector proteins can occur[3]. Ultimately, it is these effectors that allow for subversion of normal host cellular functions to the benefit of the bacterium.

Within each TTSS a conserved, class II chaperone protein is required to prevent premature association of translocator proteins while maintaining them in a secretion competent state, ready to be presented in a temporal fashion to the TTSA[4]. In the enteric pathogen *Shigella flexneri*, invasion plasmid gene (Ipg) C is the class II chaperone for invasion plasmid antigens (Ipa) B and C. Owing to this central role in supporting translocator function, IpgC is essential for *Shigella *virulence as an *ipgC *null strain is noninvasive[5]. Furthermore, upon release of the IpaB and IpaC effectors, IpgC binds to the AraC-like transcription factor, MxiE to promote expression of late-effector genes[6]. In this manner, IpgC appears to provide a critical link between TTSS induction and those events which occur immediately following bacterial contact with the host cell.

Two reports describing high-resolution crystal structures of two class II chaperones have provided a great deal of insight into the structure and function of these all alpha-helical, tetratricopeptide repeat (TPR) proteins. First, the crystal structure of an amino-terminally truncated form of SycD (SycD^21-163^) from *Yersinia enterocolitica*, was reported[7]. In this work, Büttner et al. proposed that the biologically-relevant unit of the SycD chaperone is a dimer comprised of a "head-to-head" arrangement of equivalent monomers[7]. This observation was supported by analytical gel filtration chromatography studies on targeted mutants in two residues, A61 and L65, whose disruption ablated the homomeric contacts that stabilize the dimerization interface. Moreover, a *sycD *null strain complemented with the dimerization disrupting double mutant (A61E/L65E) was unable to secrete either Yop translocator protein and exhibited characteristics typical of a *sycD *null mutant[7]. Separately, Lunelli et al. recently described the crystal structure of a carboxy-terminally truncated form of *S. flexneri *IpgC (IpgC^1-151^)[8]. Though they share only 26% sequence identity, the IpgC polypeptide displays a great deal of structural homology with SycD. However, the biological unit of this class II chaperone appeared to arise from an asymmetric dimer involving the first three alpha helices of each unique monomer. In particular, helix 1 (abbreviated hereafter as H1) and the loop connecting H1 to H2 adopted distinct arrangements in both subunits of the dimer. Deletion of the amino-terminal 21 amino acids of IpgC, which includes the entirety of H1, destroyed the dimerization ability of IpgC[8], and subsequent complementation studies demonstrated that this deleted chaperone was unable to complement an *ipgC *null strain of *Shigella *in HeLa cell invasion assays[8].

The apparent importance of asymmetric dimerization in the face of previous results that argued similarly in favor of the "head-to-head" motif suggests that both modes of dimerization may be functionally relevant. For this to be the case, however, evidence must be provided to suggest that a single class II chaperone can adopt both the asymmetric and "head-to-head" dimer arrangements. Here we present the crystal structure of an amino-terminally truncated variant (IpgC^10-155^) of the *Shigella *class II secretion chaperone IpgC. Like its full-length counterpart, we find that IpgC^10-155 ^is a dimer in solution. However, the dimerization interface of IpgC^10-155 ^observed in our crystal structure is characterized by a rotationally symmetric "head-to-head" arrangement of identical polypeptides chains. Surprisingly, this mode of quaternary structure is far more similar to that reported for SycD rather than for the longer form of IpgC (IpgC^1-151^). The potential implications for these observations to class II chaperone activity and TTSSs function are discussed.

## Results and Discussion

### Crystallization and Structure Determination of an Amino-terminally Truncated form of IpgC

**Figure 1:**
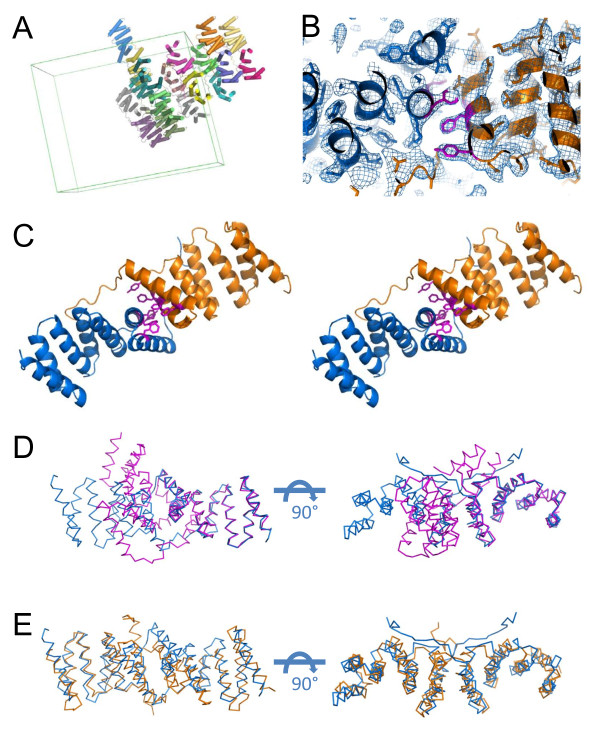
**The 3.3 Å Crystal Structure of IpgC^10-155 ^Reveals a SycD-like Dimerization Interface**. (A) Refined asymmetric unit of the IpgC^10-155 ^crystal. The 18 distinct polypeptides are colored individually; helices are depicted as cylinders for clarity. The boundaries of the primitive monoclinic cell are shown as a green box. (B) Representative model-to-map correlation for an IpgC^10-155 ^dimer pair; note that this same pair is colored identically to and further analyzed in panel C. 2Fo-Fc weighted electron density (contoured at 1.2 σ) is drawn as a blue cage. (C) Detailed stereoscopic view of the dimerization interface observed in IpgC^10-155^. The two monomers are colored in blue and orange, respectively, while the intercalated network of aromatic sidechains is shown in magenta. (D) Orthogonal views of superposition by Local-Global Alignment for the proposed IpgC^1-151 ^dimer (magenta)[8] with that of IpgC^10-155 ^(blue). (E) Orthogonal views of superposition by Local-Global Alignment for the proposed SycD^21-163 ^dimer (orange)[7] with that of IpgC^10-155 ^(blue) dimer structure. Additional quantitative descriptions of the quality of the superpositions shown in panels D and E may be found in Table 2.

Limited subtilisin treatment of recombinant, full-length IpgC was used to generate protease stable fragments of the *Shigella *chaperone that were characterized by LC-MS/MS[9]. Two predominant products were identified that corresponded to loss of nine (IpgC^10-155^) and twenty (IpgC^21-155^) residues, respectively, from the amino terminus of IpgC (Data Not Shown). Recombinant forms of both proteins were overexpressed, purified, and crystallized by hanging-drop vapor diffusion. X-ray diffraction data were collected from both crystal systems as described in Table 1. While both crystals diffracted X-rays to moderate resolution, the IpgC^21-155 ^crystals displayed very large cell parameters and were not pursued further. Initial attempts to solve the IpgC^10-155 ^crystal by molecular replacement all failed when either (a) the complete IpgC^1-151^dimer, (b) residues 10-151 from either monomer of the IpgC^1-151 ^structure, or (c) residues 20-151 from either monomer of the IpgC^1-151 ^structure were used as a search model. In contrast, iterative rounds of likelihood-based molecular replacement using a search model of residues 30-151 of a single IpgC^1-151 ^polypeptide chain eventually provided sufficient information to identify all 18 copies of the IpgC^10-155 ^protein present. Given the complexity of the asymmetric unit, and the resolution limits of the diffraction data available, a complete set of 17 strict non-crystallographic symmetry operators was used in all steps of model building and refinement. This resulted in the completed structure (Figure 1A, B and Table 1) with R_work _and R_free _values of 25.9% and 29.6%, respectively.

**Table 1:** X-ray Diffraction Data and Refinement Statistics

Diffraction Data			
Crystal		IpgC^10-155^	IpgC^21-155^
Beamline		APS 22 - BM	APS 22-ID
Wavelength (Å)		1.000	1.000
Space Group		*P*2_1_	*P*3_1 _or *P*3_2_
Cell Constants		a = 140.50	a = 86.11
		b = 71.47	b = 86.11
		c = 171.01	c = 476.27
		ß = 93.86°	
Resolution (Å)		50-3.30	50-3.40
Completeness (%)		88.5 (49.6)	89.3 (79.1)
Total Reflections		151,044	89,308
Unique Reflections		45,608	48,191
Redundancy		3.4	1.9
R_merge_(%)^a^		14.9 (50.7)	10.0 (33.2)
I/σ		7.3 (1.4)	7.0 (1.8)
			
			
Refinement			
R_work_/R_free_^b^		25.9/29.6	
B factor (Å^2^)		98.23	
RMSD			
	Bond Length (Å)	0.011	
	Bond Angle (°)	1.29	
	Dihedral Angle (°)	19.67	
Ramachandran			
	Favored (%)	91.50	
	Allowed (%)	5.80	
Protein atoms		20,070	

^a^*R*_merge _= Σ_*h*_Σ_*i*_|*I_i_*(*h*)-<*I*(*h*)>|/Σ_*h*_Σ_*i*_*I*_*i*_(*h*), where *I_i_*(*h*) is the *i*th measurement of reflection *h *and <*I*(*h*)> is a weighted mean of all measurements of *h*.

^b^*R *= Σ_*h*_|*F*_obs_(*h*)-*F*_calc_(*h*)|/Σ_*h*_|*F*_obs_|. *R*_cryst _and *R*_free _were calculated from the working and test reflection sets, respectively. The test set constituted 5% of the total reflections not used in refinement.

### Evidence for Alternative Quaternary Structures in IpgC

**Figure 2:**
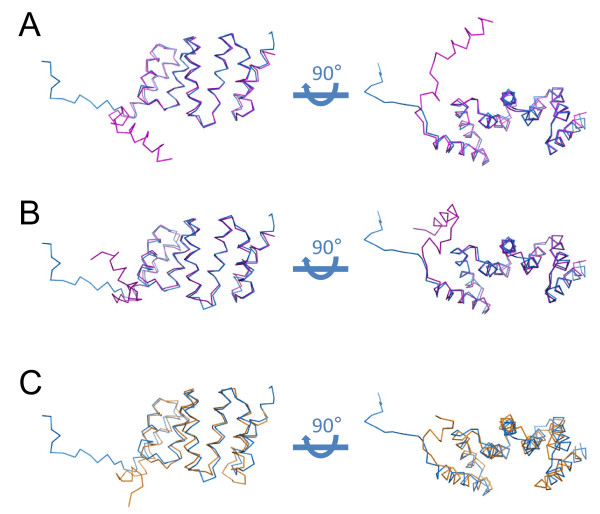
**Superposition of Monomers from Class II Chaperone Structures**. (**A**) Orthogonal views of superposition by Local-Global Alignment for the IpgC**^10-155 ^**monomer (blue) with chain A from the published structure of IpgC**^1-151 ^**(magenta)[8]. (B) Orthogonal views of superposition by Local-Global Alignment for the IpgC**^10-155 ^**monomer (blue) with chain B from the published structure of IpgC**^1-151 ^**(magenta)[8]. (C) Orthogonal views of superposition by Local-Global Alignment for the IpgC**^10-155 ^**monomer (blue) with chain A of the published structure of SycD**^21-163 ^**(orange)[7]. Additional quantitative descriptions of the quality of each superposition may be found in Table 2

As was expected from the absolute level of sequence identity between the IpgC^10-155 ^structure presented here and either IpgC^1-151 ^monomer described by Lunelli et al.[8], the IpgC polypeptide monomers superimpose well with one another (Figure 2A, B and Table 2). The only noteworthy difference between these structures lies in the amino terminal regions (i.e. residues 1-30) of these two forms of IpgC. Whereas these residues in IpgC^1-151 ^form two short, ordered alpha helices[8], the same region of IpgC^10-155 ^adopts an extended coil-like structure. This significant structural difference provides the most likely explanation for why molecular replacement searches with either IpgC^1-151 ^dimers or models with insufficiently truncated amino termini failed. Separately, quantitative comparisons of structure superposition[10] between IpgC^10-155 ^and SycD^21-163 ^reveal that the corresponding monomer structures are nearly as similar to one another as are the two forms of IpgC (Figure 2C and Table 2). While this is somewhat surprising given the limited sequence identity (~26%) between these proteins, it further underscores the high level of tertiary structure conservation among TTSS class II chaperones.

**Table 2:** Superposition Analysis for Selected Monomers and Dimers as Determined by Local-Global Alignment

Structure 1	Structure 2	Corresponding Cα positions^a^	RMSD (Å)	Sequence Identity (%)	LGA_S^c^
IpgC^1-151 ^(chain A)	IpgC^10-155^	122/137	0.80	99.18^b^	88.32
IpgC^1-151 ^(chain B)	IpgC^10-155^	123/137	1.01	100.00	87.62
SycD^21-163^ (chain A)	IpgC^10-155^	120/137	1.56	25.83	80.61
SycD^21-163 ^(AU)	IpgC^10-155 ^(chains A&B)	199/274	2.34	18.59	51.27
IpgC^1-151 ^(AU)	IpgC^10-155 ^(chains A&B)	135/274	1.35	93.33	48.14

^a^Denotes the number of residues from structure 1 that superimpose within 4.0 Å distance of an equivalent position in structure 2.

^b^Even though the actual sequence identity between these two samples is 100%, the Cα location of A30 in chain of A of the IpgC^1-151 ^structure actually lies closer to that of I28 of IpgC^10-155 ^when these two structures are superimposed.

^c^The LGA_S parameter represents a scoring function to evaluate the overall levels of structural similarity between two sets of coordinates. For each set of corresponding residues, it combines information pertaining to both the faction of residues that overlap within a given RMSD window as well as those that overlap within a given distance cutoff[10].

Examination of the refined structure indicates that the IpgC^10-155 ^crystals consist of an ordered lattice of protein dimers. However, much like the structures of SycD^21-163^[7], IpgC^1-151^[8], and PcrH^21-160^[11], more than one plausible dimerization arrangement are observed. The first class of dimer is present in nine crystallographically unique copies and is characterized by a "head-to-head" orientation, where each monomer is related by a single axis of rotational symmetry (Figure 1C and Additional file 1, Figure S1). This "head-to-head" dimer buries an average of 1262.5 Å^2 ^of surface area upon formation, which compares favorably to the 1381.9 Å^2 ^interface previously described for IpgC^1-151^[8]. A distinct class of rotationally-symmetric arrangement occurs only once within the asymmetric unit, though it can be generated for all other IpgC^10-155 ^chains by application of crystallographic operators (Additional file 2, Figure S2). It is worth noting, however, that this "tail-to-tail" dimer buries only an average of 756.5 Å^2 ^upon formation, or approximately 60% of the surface masked by the "head-to-head" structure. Furthermore, the contacts present in the "head-to-head" dimer are more extensive and conserved to a far greater extent (21 of 40 residues, or roughly 53%) than are those found in the "tail-to-tail" structure (6 of 18 residues, or roughly 33%). When considered together, these data strongly suggest that the "head-to-head" dimer is the relevant structure for IpgC^10-155^, and that the other dimerization mode most likely arises from crystallization.

The identification of a rotationally symmetric dimer within the IpgC^10-155 ^crystal raised questions about its relationship to the dimers previously described for TTSS class II chaperones (Figure 1D, E and Table 2). In this regard, the "head-to-head" arrangement of IpgC^10-155 ^superimposes relatively poorly with the asymmetric dimer of IpgC^1-151^; overall, only 135 of 274 corresponding Cα positions lie within 4.0 Å distance and an RMSD of 1.35 Å. By contrast, the IpgC^10-155^dimer overlays surprisingly well with the dimer previously described for SycD^21-163^; here, 199 of 274 corresponding Cα positions lie within 4.0 Å distance with an RMSD of 2.34 Å. It is important to note that the lower RMSD for the first superposition arises because Cα positions that lie outside of the 4.0 Å distance cutoff are omitted from RMSD calculation. Thus, even though the two IpgC structures share a substantially higher level of identity in terms of sequence and overall monomer structure (Figure 2 and Table 2), the far greater number of aligned residues between the IpgC^10-155 ^and SycD^21-163 ^dimers indicates that their "head-to-head" quaternary structures are closely related.

Although the biological unit of the IpgC^1-151 ^structure has been defined as an asymmetric dimer of structurally unique subunits, examination of IpgC^1-151 ^chains related by crystallographic symmetry reveals two separate contacts reminiscent of the IpgC^10-155 ^quaternary structure (Additional file 3, Figure S3). The first of these contacts is found in the IpaB peptide-bound form of IpgC^1-151^, and buries 666.9 Å^2 ^of surface area upon formation (Additional file 3, Figure S3A). When compared carefully to the IpgC^10-155 ^dimer, it is apparent that the symmetry-related IpgC^1-151 ^chain in this contact is rotated to a greater extent relative to the monomer found within the asymmetric unit; as a result, only 130 of 264 Cα positions superimpose within 4.0 Å distance. Separately, a "head-to-head" dimer is also observed within the lattice contacts of the unbound IpgC^1-151 ^structure (Additional file 3, Figure S3B). This arrangement buries 950.4 Å^2 ^of surface area upon formation and, aside from the previously mentioned difference in the amino terminal region, shares a higher level of homology to the IpgC^10-155 ^dimer. In this case, 176 of 264 possible Cα positions superimpose within 4.0 Å distance. Most importantly, the aromatic residues which line this interface are identical to those found in the IpgC^10-155 ^dimer (Additional file 3, Figure S3C), as described below.

### Residues that Comprise the 'Head-to-Head' Dimerization Interface in IpgC are Conserved Across TTSS Class II Chaperones

While the IpgC^10-155 ^dimer buries approximately 1260 Å^2 ^of surface area upon formation, closer inspection reveals that this interaction is comprised largely of two separate regions from each respective monomer. The first of these gives rise to the SycD-like interface (~680 Å^2^), and involves an intricate array of almost exclusively hydrophobic interactions between the α2 and α3 helices of opposing IpgC^10-155 ^chains. Chief among these is a network of homophilic contacts between Phe residues at positions 46, 58, and 61, whose sidechains nearly intercalate with one another (Figure 1B, C). Hydrophobic interactions aside, a single hydrogen bond between the sidechains of Tyr^42 ^and Glu^53 ^is also found within this interface. Separately, a distinct region of contact that masks nearly 580 Å^2 ^of surface area is likewise observed in the IpgC^10-155 ^dimer. This interface arises from packing of nearly the entire extended amino terminus of one IpgC^10-155 ^chain against primarily the α5 helix of its counterpart polypeptide (Additional file 4, Figure S4). Intriguingly, this region includes residues Ala^94 ^and Val^95^, two residues whose concerted mutation has been shown to disrupt the dimerization of IpgC^1-151^[8]. Analytical gel filtration chromatography was used to analyze the effect of this double mutant on the oligomeric state of both IpgC^1-151 ^and IpgC^10-155 ^(Additional file 5, Figure S5). However, both proteins migrated as a single species with an observed molecular weight of approximately 40 kDa in this assay (Additional file 6, Figures S6). In contrast to previous data[8], these results suggest that dimerization may not be fully ablated by simultaneous mutation of both Ala^94 ^and Val^95^.

**Figure 3:**
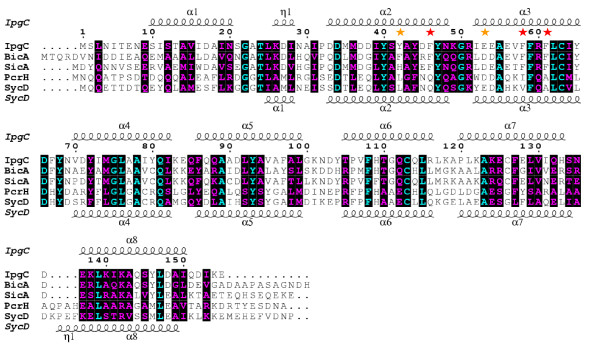
**Residues that Comprise the Head-to-Head Dimerization Interface in IpgC^10-155 ^are Conserved among Class II Chaperones**. The sequences of IpgC and its homologs were aligned using the ClustalW method[21] and compared to the secondary structure elements of both the IpgC**^1-151 ^**[8] and the SycD**^21-163^**[7] crystal structures with Espript[22]. Residue positions were numbered using the IpgC sequence as a reference. Identical residues are shown in cyan typeface, while similar residues (as judged by the BLOSUM62 substitution matrix) are shown in purple typeface. Residues at positions 46, 58, and 61 form "head-to-head" contacts in the crystal structure (see Figure 1C) and are denoted with a red star. Residues at positions 42 and 53 appear to form sidechain-to-sidechain hydrogen bonds and are denoted with an orange star.

The nature and extent of the contacts made by the IpgC^10-155 ^amino terminus makes it difficult to interpret the overall significance of sequence conservation in this region of the protein. However, examination of a structure-based multiple-sequence alignment of class II chaperones indicates that nearly all of the positions mentioned in regard to the SycD-like interface are well conserved (Figure 3). This is especially true in the case of BicA and SicA, which are less divergent members of this family. It is somewhat surprising that the residues which participate in IpgC^10-155 ^homophilic contacts are biochemically distinct from those of SycD, which instead relies on hydrophobic contacts of opposing Ala^61 ^and Leu^65 ^sidechains for dimerization[7]. The fact that the quaternary arrangement of IpgC^10-155 ^and SycD^21-163 ^is so similar even though the residues lining this interface are different strongly suggests that the ability to form this "head-to-head" dimer is a conserved structural feature among TTSS class II chaperones.

### The 'Head-to-Head' Dimer of IpgC Supports Chaperone Activity

**Figure 4:**
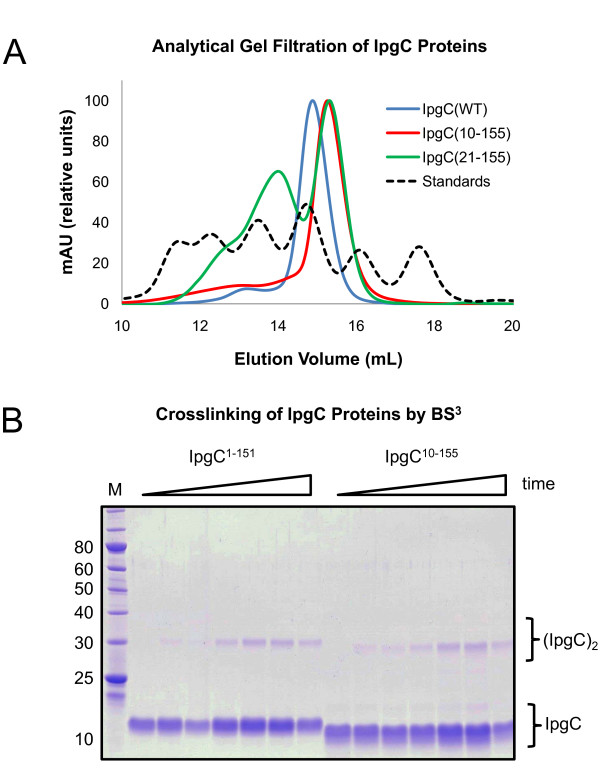
**IpgC^10-155 ^Exists as a Dimer in Solution**. (A) Samples of purified IpgC (5 mg/mL) proteins were injected onto an analytical gel-filtration column and the elution profiles were compared to a series of known standards to derive an estimation of protein molecular weight (see Additional file 6, Figure S6). The sample identities are IpgC**^WT ^**(blue), IpgC**^10-155 ^**(red), and IpgC**^21-155 ^**(green). The standard mixture is shown as a black dashed line. Aside from the standard injection, all curves were normalized to a maximum peak height of 100 mAU for clarity. (B) Purified samples of IpgC representing both asymmetric (IpgC**^1-151^**) and "head-to-head" dimers (IpgC**^10-155^**) were exposed to the amine-reactive crosslinking agent BS**^3^**. Samples were removed at 0, 15, 30, 60, 90, 120, and 240 min following the start of crosslinking, quenched by incubation with Tris, and analyzed for the presence of covalent dimers by SDS-PAGE.

**Figure 5:**
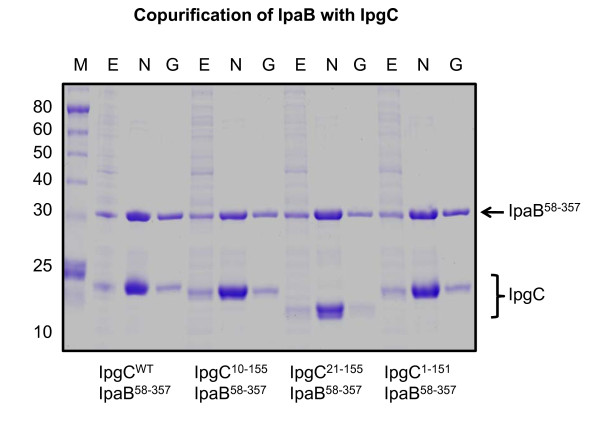
**Amino-terminally Truncated forms of IpgC Retain Chaperone Activity for IpaB**. Full-length and various truncated forms of IpgC were coexpressed with IpaB**^58-357 ^**in recombinant strains of ***E. coli***. Following induction of protein expression, cells were harvested, lysed, and the soluble extracts thereof were subjected to a copurification protocol to assess the ability of IpgC to bind IpaB. SDS-PAGE was used to analyze various protein samples following each step of copurification: M, protein molecular weight standards; E, clarified cellular extract following microfluidization and centrifugation; N, pooled elute following Ni**^2+^**-Sepharose chromatography; G, pooled fractions of the complex following gel-filtration chromatography. Since IpaB accumulates in inclusion bodies in the absence of IpgC activity[5], and since the untagged IpaB lacks the ability to bind Ni**^2+^**-NTA Sepharose on its own[20], all four IpgC proteins used in this study maintain chaperone activity.

The remarkably different mode of dimerization between IpgC^10-155 ^and IpgC^1-151 ^raised questions as to whether two distinct quaternary arrangements could be supported by this protein. To address this question, analytical gel filtration chromatography was used to determine whether the dimers observed in the IpgC^10-155 ^structure were present in solution. Comparison of the elution profile of IpgC^10-155 ^to a calibration curve of globular protein standards reveals an observed molecular weight of approximately 36 kDa (Figure 4A and Additional file 6, Figure S6); given a theoretical value of 16.7 kDa per monomer, this strongly suggests that this amino-terminally truncated IpgC behaves as a dimer in solution. This conclusion is also supported by chemical crosslinking of IpgC^10-155^, which reveals the generation of covalent IpgC dimers following a timecourse exposure to the amine reactive agent BS^3 ^(Figure 4B). Previous studies have also reported that genetic truncation of residues 1-20 in IpgC results in the formation of soluble but aggregated protein and that this is most likely due to the loss of both helices responsible for asymmetric dimerization of IpgC[8]. However, two pieces of evidence suggest that this may not be entirely the case. First, while recombinant IpgC^21-155 ^did not behave as a single, ideal species when analyzed by gel filtration, it nevertheless migrated according to the defined oligomeric states of tetramer (63 kDa) and dimer (35 kDa). Second, IpgC^21-155 ^yielded diffraction quality crystals (Table 1), an outcome that seems highly unlikely with an aggregated sample. Separately, both of these amino-terminally deleted forms of IpgC maintain the ability to bind IpaB within the context of a copurification assay (Figure 5). Since IpaB cannot be expressed in a soluble fashion in *E. coli *without its cognate chaperone[5], this result indicates that these amino-terminally deleted proteins maintain the chaperone activity previously attributed to IpgC[12]. Together, these results strongly suggest that residues 1-20 are dispensable for both dimerization of IpgC and for chaperone activity, and that IpgC is capable of adopting both asymmetric and "head-to-head" dimer arrangements in solution.

## Conclusions

In the last year, two separate studies have reported the crystal structures of class II chaperones bound to peptide fragments of TTSS translocator proteins. Even though the structures of IpgC^1-151^-IpaB[8] and PcrH^21-160^-YopD[11] are meant to mimic recognition of separate classes of full-length translocator proteins from two distinct organisms, both structures reveal that the translocator peptide lies within a groove found on the concave TPR "hand" of the chaperone. This indicates that their mechanism of translocator/ligand recognition is similar, despite the fact that the quaternary structures appear to differ considerably between IpgC^1-151 ^and PcrH^21-160^. The significant differences between the "head-to-head" dimer observed in both SycD^21-163^[7] and PcrH^21-160^[11] with the asymmetric structure of IpgC^1-151 ^raise important questions regarding the precise nature of class II chaperone dimers in the physiological setting. Further complicating this issue are the cogent biophysical, biochemical, and/or functional data which support each of these crystal structures.

Our observation that a single TTSS chaperone can adopt two distinct quaternary arrangements suggests that both the asymmetric and head-to-head dimers may have important physiological roles in bacterial TTSSs. As stated earlier, IpgC has the ability to bind two separate translocator proteins, IpaB and IpaC[12], as well as the AraC-family transcription factor, MxiE[13]. The ability of IpgC to bind each of these proteins is regulated by the secretion state of the *S. flexneri *cell[1]. Secretion of both IpaB and IpaC through the TTSA needle liberates IpgC, and allows it to interact with MxiE; this culminates in the expression of the late effectors[6]. It is believed that an amino terminal secretion signal targets effectors to the secretion system and that chaperones may also be involved in guidance of their complexes to the base of the TTSA needle[6]. The potential switch in quaternary structure of IpgC may therefore be involved with its ability to effectively bind or deliver translocators to the secretion system. For example, though both types of chaperone dimer are competent to bind peptide mimics of their translocator targets, a change in dimerization state might alter the stoichiometry of various chaperone/ligand complexes within the context of full-length proteins. Addressing this possibility will require more thorough characterization of translocator proteins, such as IpaB and IpaC, for which little tertiary structural information is currently available. Along these lines, IpgC could also transition between asymmetric and symmetric dimerization modes to accommodate its broad range of interaction partners. Because the change in dimerization appears to correlate with a loss of amino acids at the amino terminus of IpgC, an ordered proteolytic event in this region might trigger a change in quaternary structure that affects IpgC function. Whether such a transition could result in a change in the role of IpgC from secretion chaperone to transcriptional coactivator remains to be determined. In any case, additional study will be needed to explore the potential roles of both modes of dimerization in the *Shigella *TTSS as well as that from other pathogens.

## Methods

### Cloning, overexpression and purification of recombinant forms of IpgC

A designer gene fragment encoding residues 10-155 of IpgC was amplified from the virulence plasmid of *Shigella flexneri *via PCR and subcloned into pT7HMT[14]. Following confirmation of its DNA sequence, this expression vector was transformed into BL21 (DE3) *Escherichia coli *cells and cultured in Terrific Broth at 37°C to an A_600 nm _of 0.8. Protein expression was induced overnight at 18°C by adding IPTG to 1 mM final concentration. Cells were harvested by centrifugation, resuspended in lysis buffer (20 mM Tris pH 8.0, 500 mM NaCl, and10 mM imidazole), and then lysed in a microfluidizer. The soluble target protein was collected in the supernatant following centrifugation of the cell homogenate and purified on a Ni^2+^-NTA Sepharose column according to standard protocols[14]. Recombinant TEV protease was used to digest the fusion affinity tag from the target protein. After desalting into 20 mM Tris (pH 8.0), final purification was achieved by Resource Q anion-exchange chromatography (GE Biosciences). Following this, the purified protein was concentrated to 10 mg/mL and exchanged into H_2_O for further use. A similar protocol was used to subclone, overexpress, and purify full-length IpgC, a further truncated form that consisted of residues 21-155 (IpgC^21-155^), and the IpgC^1-151 ^variant described by Lunelli et al.[8]. Expression vectors encoding the Ala^94^Glu/Val^95^Gln double mutant of both IpgC^10-155 ^and IpgC^1-151 ^were generated by PCR using the two-step megaprimer method[15]; the corresponding proteins were overexpressed and purified as described above.

### Crystallization

IpgC^10-155 ^was crystallized by vapor diffusion of hanging drops at 20°C. Specifically, 1 μL of protein solution (10 mg/mL in ddH_2_O) was mixed with 1 μL of reservoir solution containing 0.2 M magnesium chloride hexahydrate, 0.1 M Bis-Tris (pH 6.5) and 25% (w/v) PEG 3350, and the drops were equilibrated over 500 μL of reservoir solution. Clusters of needle-shaped crystals appeared overnight and continued to grow in size for approximately 7 days. Mechanical disruption of these clusters was used to obtain single, diffraction quality samples for diffraction analysis. Crystals were flash cooled in a cryoprotectant solution consisting of reservoir buffer with an additional 5% (w/v) PEG 3350. Crystals of IpgC^21-155 ^were also produced using an analogous approach. Briefly, 1 μL of protein solution (10 mg/mL in ddH_2_O) was mixed with 1 μL of reservoir solution containing 0.1 M HEPES (pH 7.5) and 2.0 M ammonium formate, and the drops were equilibrated over 500 μL of reservoir solution. Single diamond shaped crystals appeared overnight and continued to grow for 2-3 days. Crystals were flash cooled in a cryoprotectant solution consisting of reservoir buffer with 30% (v/v) glycerol. Diffraction quality crystals were not obtained for full-length IpgC.

### Structure determination, refinement and analysis

Monochromatic X-ray diffraction data (γ = 1.000 Å) were collected from single IpgC^10-155 ^and IpgC^21-155^crystals at 100 K using beamlines 22-BM and 22-ID, respectively, of the Advanced Photon Source, Argonne National Laboratory (Table 1). Following data collection, individual reflections were indexed, integrated, and scaled using HKL2000[16]. Initial phase information was obtained for the IpgC^10-155 ^data by maximum-likelihood molecular replacement using PHASER[17]. Residues 30-151 of a single copy of the refined IpgC^1-151 ^structure were used as a search model[8]. The single most highly scored solution contained 18 unique IpgC^10-155 ^polypeptides in the asymmetric unit, which corresponded to a solvent content of 56.8%.

Structure refinement was carried out using the protocols implemented in phenix.refine[18]. First, three rounds of individual coordinate and isotropic atomic-displacement factor refinement were conducted and the refined model was used to calculate both 2Fo-Fc and Fo-Fc maps. These maps were used to manually build residues 18-29 and 152-154 of the master polypeptide chain, which is denoted chain A in the PDB file. This intermediate model was subjected further to an identical series of refinement steps prior to a final, single round of TLS refinement in phenix.refine; each individual polypeptide chain was treated as its own unique TLS group. The final model displays R_work_/R_free _values of 25.9/29.6%, respectively, and consists of residues 18-154 for all 18 copies of IpgC^10-155 ^present in the asymmetric unit. RAMPAGE analysis of the final model revealed that 91.5% and 5.8% of the 2,430 residues modeled occupied either favored or allowed regions of the Ramachandran plot, respectively[19]. Additional electron density that corresponded to N terminally directed residues were visible in both 2Fo-Fc and Fo-Fc maps calculated from the final model. Side chain features were poor in these areas, however, and this precluded accurate modeling of these residues in the final structure. The coordinates of the crystal structure described here have been deposited in the RCSB database under the accession code 3KS2.

### Analytical gel filtration chromatography

Purified protein samples (5 mg/mL) were separated on a Tricorn Superdex 200 10/300 analytical gel filtration chromatography column (GE Biosciences) that had been previously equilibrated in a buffer of 20 mM Tris-HCl (pH 8.0), 200 mM NaCl, 1 mM DTT at 4°C. Estimates of molecular weight and oligomerization were made by comparing the retention time of individual samples to those of globular protein standards (Bio-Rad).

### Chemical crosslinking

Bis(Sulfosuccinimidyl) suberate (BS^3^; 80 μL of a 250 μM solution in ddH_2_O) was added to 20 μL samples (2 mg/mL) of purified IpgC^1-151 ^and IpgC^10-155 ^at 20°C. 5 μL aliquots from each reaction were withdrawn at various time points over the course of 240 min and excess BS^3 ^was quenched by adding 0.75 μL of 25 mM Tris (pH 8.0) for 30 min. Samples were analyzed under reducing conditions by electrophoresis (10% SDS-PAGE) using a Tris-Tricine buffer system.

### Copurification assay for chaperone activity

Chaperone activity of full-length IpgC and various deletion proteins was monitored by chromatographic copurification. Specifically, a designer gene fragment encoding a protease-stable domain of *S. flexneri *IpaB (residues 58-357; denoted IpaB^58-357^) was generated by PCR, subcloned in the expression vector pACYC-Duet (Novagen), and sequenced; this vector provides for expression of IpaB^58-357 ^without any fusion tag, and IpaB does not bind significantly to Ni^2+^-NTA Sepharose on its own accord[20]. The resulting plasmid was co-transformed with various pT7HMT-IpgC expression vectors (described above) into *E. coli *BL21(DE3) cells. Cotransformants were identified by antibiotic selection with both chloramphenicol and kanamycin. Cells harboring both expression vectors were cultured and protein expression was induced according to standard methods. Homogenates of induced cells (250 mL total culture volume) were prepared by microfluidization, clarified by centrifugation, and subjected to Ni^2+^-NTA Sepharose chromatography as described above. Following this, the crude eluate was further separated on a Superdex 75 26/60 preparative gel-filtration column (GE Biosciences). Samples were analyzed under reducing conditions by 4-10% gradient SDS-PAGE using a Tris-Tricine buffer system.

### Miscellaneous

Multiple sequence alignments were carried out using CLUSTALW[21] and aligned with secondary structure elements using ESPRIPT[22]. Sequences used in alignment, along with their respect accession numbers, were as follows: *Shigella flexneri *IpgC (GI:32307022), *Burkholderia pseudomallei *BicA (GI:126447932), *Salmonella typhimurium *SicA (GI:975294), *Pseudomonas aeruginosa *PcrH (GI: 29826004) and *Yersinia enterocolitica *SycD (GI:23630571). Three-dimensional structures were analyzed using the Protein Interfaces, Surfaces, and Assemblies server (PISA) [23] and superimposed using the Local-Global Alignment method (LGA)[10]. Representations of all structures were generated using PyMol[24].

## Abbreviations

(IpgC): Invasion plasmid gene C; (SycD): Specific Yersinia chaperone D; (TTSS): Type III Secretion System; (TTSA): Type III Secretion Apparatus; (IpaB): Invasion plasmid antigen B; (IpaC): Invasion plasmid antigen C; (MxiE): Membrane expression of ipa E; (TPR): Tetratricopeptide repeat; (LC-MS): Liquid Chromatography Mass Spectrometry; (TEV): Tobacco etch virus; (IPTG): Isopropyl β-D-1-thiogalactopyranoside; (SicA): Salmonella invasion chaperone A; (BicA): Burkholderia invasion chaperone A.

## Authors' contributions

MLB identified and optimized the crystallization conditions, completed the X-ray diffraction studies, and performed the gel filtration and co-purification assays. LZ constructed the expression vectors and assisted with functional analysis. MLB and BVG solved and analyzed the structure. MLB, WLP, and BVG wrote the manuscript. WLP and BVG supervised and coordinated the study. All authors read and approved the final manuscript.

## Supplementary Material

Additional file 1 **Analysis of All Dimer Pairs Found in the IpgC^10-155 ^Asymmetric Unit**. All nine IpgC^10-155 ^dimer pairs were superimposed by Local-Global Alignment to examine their overall similarity to one another. (A) Two orthogonal stereoscopic views of each dimer pair superimposed. A legend describing the identity of each protein chain in the corresponding PDB entry (accession code 3KS2) is shown underneath. (B) Quantitative analysis of all dimer superpositions **from panel A **presented in Table format. Click here for file

Additional file 2 **Comparison of Alternative Dimer Assemblies in the IpgC^10-155 ^Crystal**. Potential contacts between either non-crystallographic or crystallographic symmetry-related IpgC**^10-155 ^**chains were evaluated using the EBI Protein Interfaces Surfaces and Assemblies (PISA) server[23]. (A) Two views of the rotationally-symmetric "head-to-head" dimer (as shown in Figure 1B, C) that is found in nine copies within the IpgC**^10-155 ^**asymmetric unit. On average, this arrangement buries 1262.5 Å**^2 ^**of surface area upon formation. (B) Two views of a rotationally-symmetric "tail-to-tail" dimer found in nine copies following application of crystallographic symmetry operators. On average, this arrangement buries 756.5 Å**^2 ^**of surface area upon formation. For the sake of clarity, the relative orientation of the orange colored IpgC**^10-155 ^**chain is identical between panels A and B. Click here for file

Additional file 3 **Comparison of IpgC^10-155 ^Dimers to Head-to-Head Dimer Assemblies Present in IpgC^1-151 ^Crystals**. Both the free (3GYZ) and IpaB peptide-bound (3GZ1) structures of IpgC^1-151 ^were examined for potential "head-to-head" dimerization contacts similar to those observed in the IpgC^10-155 ^structure presented in Figure 1. (A) Two orthogonal views of a putative dimer (magenta and cyan) from 3GZ1 superimposed with a prototypic dimer of IpgC^10-155^(blue and orange). This dimer is generated by applying the crystallographic symmetry operator *x,-y+1,-z*, and buries 666.9 Å^2 ^of surface area upon formation. (B) Two orthogonal views of a putative dimer from 3GYZ superimposed with the IpgC^10-155 ^dimer; all chains are colored as in panel A. This dimer is generated by applying the crystallographic symmetry operator *x-y,-y,-z+2/3*, and buries 950.4 Å^2 ^of surface area upon formation. (C) Two identical views of the IpgC^10-155 ^dimer (left) and the symmetry-generated dimer from 3GYZ shown in panel B (right). The set of aromatic residues described in Figure 3 are colored magenta and blue in the left and right panels, respectively. Click here for file

Additional file 4 **Magnified View of the Interface between the Amino-terminal Region and Helix α5 in an IpgC^10-155 ^Dimer**. The individual monomers that comprise the IpgC**^10-155 ^**dimer are depicted as blue and orange cartoons, while the residues contributing to buried surface area (ball-and-stick) in this region are colored in magenta and cyan, respectively. Residues Ala**^94 ^**and Val**^95^**, which are critical for dimerization in IpgC**^1-151 ^**[8], are colored yellow for reference. (A) Rendering of the IpgC**^10-155 ^**dimer from a viewing plane opposite that of Figure 1B, C. (B) Rotated view of panel A such that the axis of helix α5 is orthogonal to the plane of the page. Note the packing of the amino-terminal residues (magenta) against the sidechains of α5 (cyan and yellow). (C) Similar to panel B, but the viewing plane has been rotated 90° with respect to the page. The zigzag nature of the packing between the magenta-colored amino-terminus and the opposing monomer is evident in this image. Click here for file

Additional file 5 **IpgC Double Mutants Exist as Dimers in Solution**. Description: Samples of purified IpgC (5 mg/mL) proteins were injected onto an analytical gel-filtration column and the elution profiles were compared to a series of known standards to derive an estimation of protein molecular weight (see Additional File 6, Figure S6). The sample identities are IpgC^10-155 ^(red), IpgC^10-155 ^Ala^94^Glu/Val^95^Gln (cyan), and IpgC^1-151 ^Ala^94^Glu/Val^95^Gln (purple). The standard mixture is shown as a black dashed line. Aside from the standard injection, all curves were normalized to a maximum peak height of 100 mAU for clarity. Click here for file

Additional file 6 **Calibration of the Analytical Size Exclusion Chromatography Column**. Size exclusion standard curve where observed molecular weight is plotted as a function of elution volume. Calibration points (black diamonds) correspond to the following standard proteins: β-amylase (200 kDa), dehydrogenase (150 kDa), serum albumin (66 kDa), ovalbumin (43 kDa), carbonic anhydrase (29 kDa) and cytochrome c (12.4 kDa). Estimations of molecular weight were determined using the relationship MW = 31.195×*e*^(-0.443×(E.V.))^, where the molecular weight (MW) is given in kDa and the elution volume (E.V.) is given in mL; the correlation coefficient for this relationship (R^2^) is 0.987 (blue line). Click here for file
