# Supplementary material for: Evidence for alternative quaternary structure in a bacterial Type III secretion system chaperone
Source: BMC Struct Biol. 2010 Jul 15;10:21. doi: 10.1186/1472-6807-10-21 (PMC2912912; doi:10.1186/1472-6807-10-21)
Supplement: Additional file 1 — Analysis of All Dimer Pairs Found in the IpgC10-155 Asymmetric Unit. All nine IpgC10-155 dimer pairs were superimposed by Local-Global Alignment to examine their overall similarity to one another. (A) Two orthogonal stereoscopic views of each dimer pair superimposed. A legend describing the identity of each protein chain in the corresponding PDB entry (accession code 3KS2) is shown underneath. (B) Quantitative analysis of all dimer superpositions from panel A presented in Table format. [file 1472-6807-10-21-S1.PPT]

## Slide 1
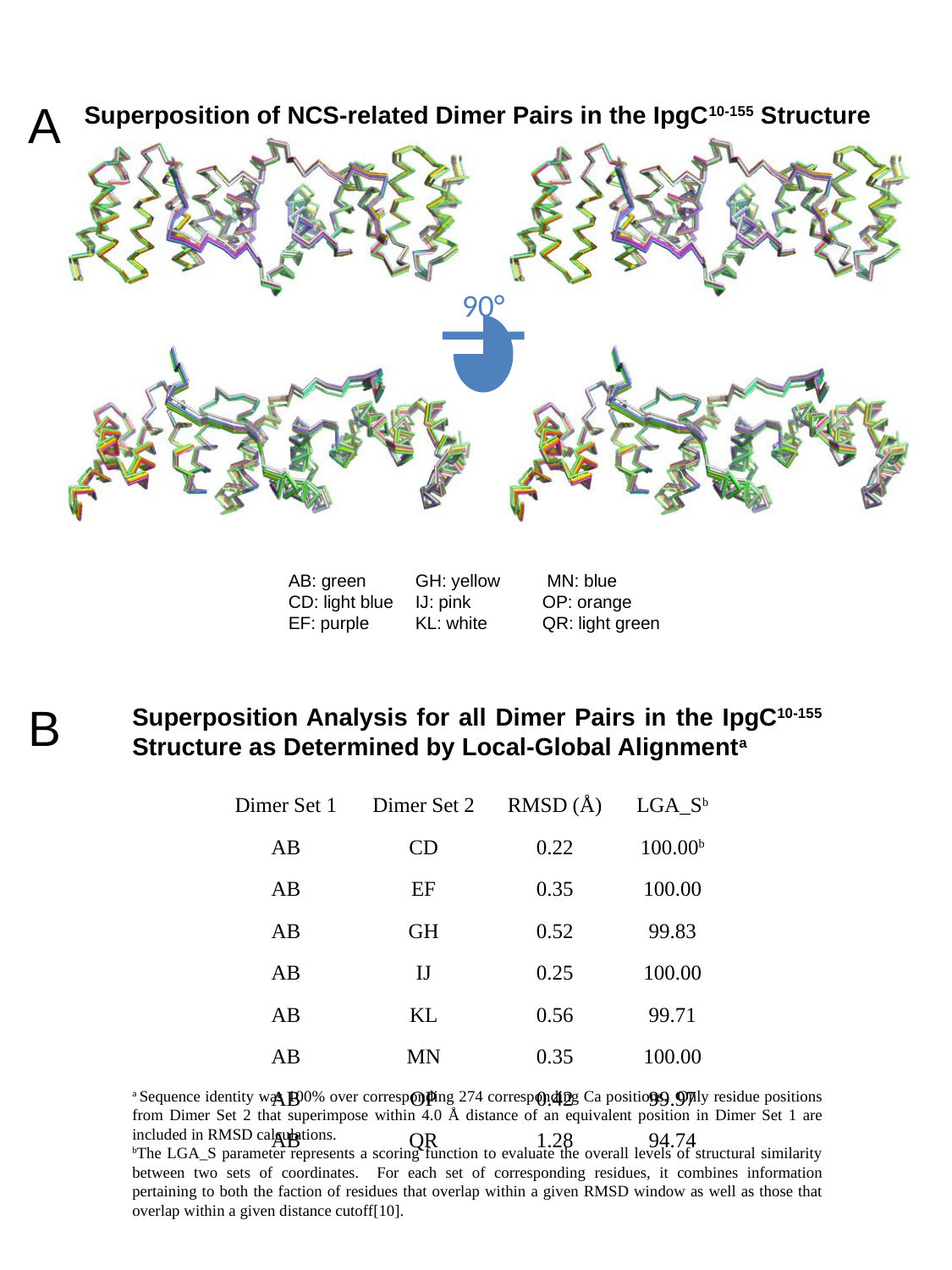

A
Superposition of NCS-related Dimer Pairs in the IpgC10-155 Structure
90°
AB: green 	GH: yellow	 MN: blue
CD: light blue 	IJ: pink 	OP: orange
EF: purple 	KL: white 	QR: light green
Superposition Analysis for all Dimer Pairs in the IpgC10-155 Structure as Determined by Local-Global Alignmenta
a Sequence identity was 100% over corresponding 274 corresponding Ca positions. Only residue positions from Dimer Set 2 that superimpose within 4.0 Å distance of an equivalent position in Dimer Set 1 are included in RMSD calculations.
bThe LGA_S parameter represents a scoring function to evaluate the overall levels of structural similarity between two sets of coordinates. For each set of corresponding residues, it combines information pertaining to both the faction of residues that overlap within a given RMSD window as well as those that overlap within a given distance cutoff[10].
B
| Dimer Set 1 | Dimer Set 2 | RMSD (Å) | LGA\_Sb |
| --- | --- | --- | --- |
| AB | CD | 0.22 | 100.00b |
| AB | EF | 0.35 | 100.00 |
| AB | GH | 0.52 | 99.83 |
| AB | IJ | 0.25 | 100.00 |
| AB | KL | 0.56 | 99.71 |
| AB | MN | 0.35 | 100.00 |
| AB | OP | 0.42 | 99.97 |
| AB | QR | 1.28 | 94.74 |
