# Supplementary figures and images for: Evidence for alternative quaternary structure in a bacterial Type III secretion system chaperone
Source: BMC Struct Biol. 2010 Jul 15;10:21. doi: 10.1186/1472-6807-10-21 (PMC2912912; doi:10.1186/1472-6807-10-21)

## Slide 1
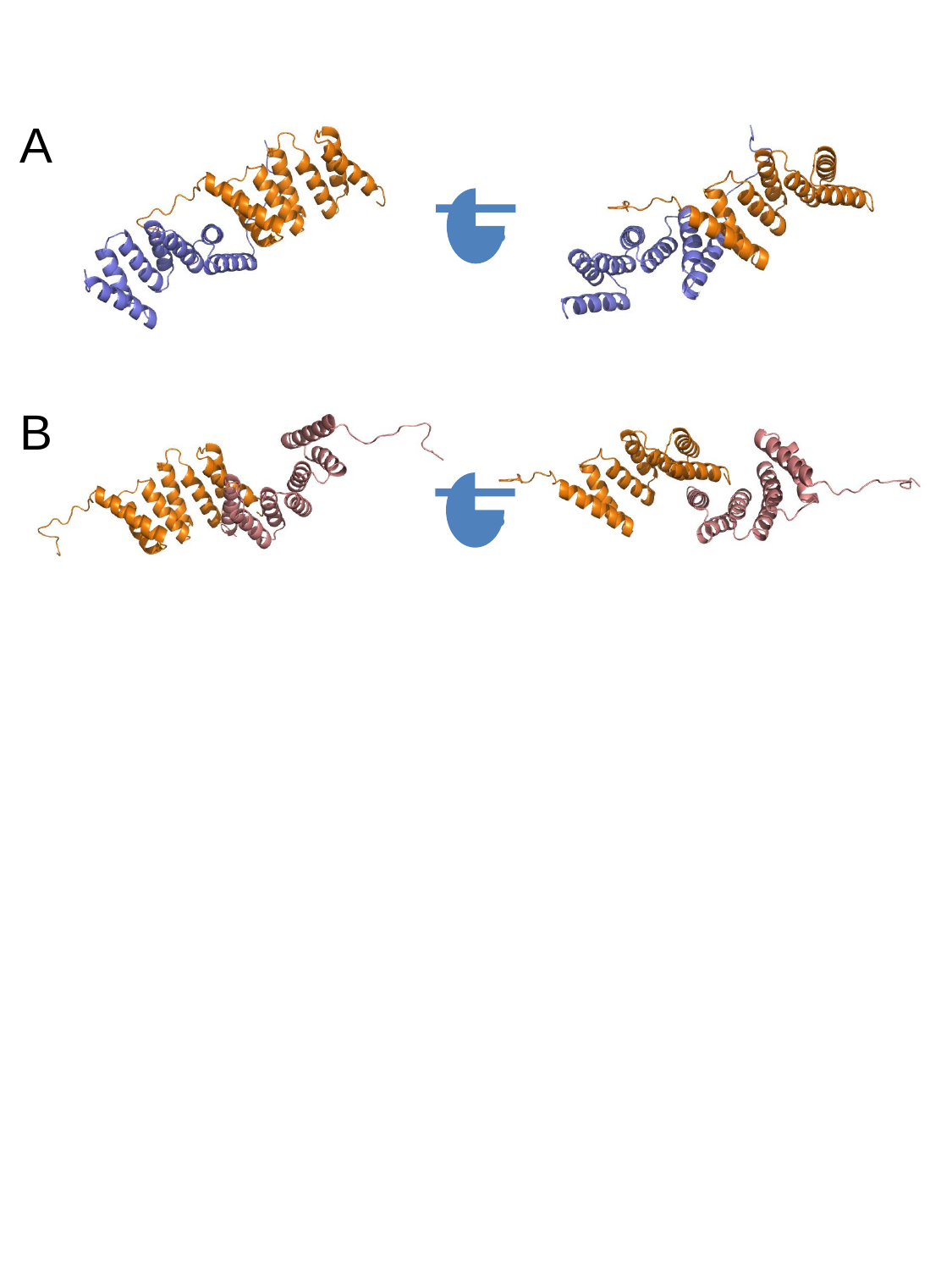

A
45°
45°
B

Supplement: Additional file 2 — Comparison of Alternative Dimer Assemblies in the IpgC10-155 Crystal. Potential contacts between either non-crystallographic or crystallographic symmetry-related IpgC10-155 chains were evaluated using the EBI Protein Interfaces Surfaces and Assemblies (PISA) server[23]. (A) Two views of the rotationally-symmetric "head-to-head" dimer (as shown in Figure 1B, C) that is found in nine copies within the IpgC10-155 asymmetric unit. On average, this arrangement buries 1262.5 Å2 of surface area upon formation. (B) Two views of a rotationally-symmetric "tail-to-tail" dimer found in nine copies following application of crystallographic symmetry operators. On average, this arrangement buries 756.5 Å2 of surface area upon formation. For the sake of clarity, the relative orientation of the orange colored IpgC10-155 chain is identical between panels A and B. [file 1472-6807-10-21-S2.PPT]

## Slide 1
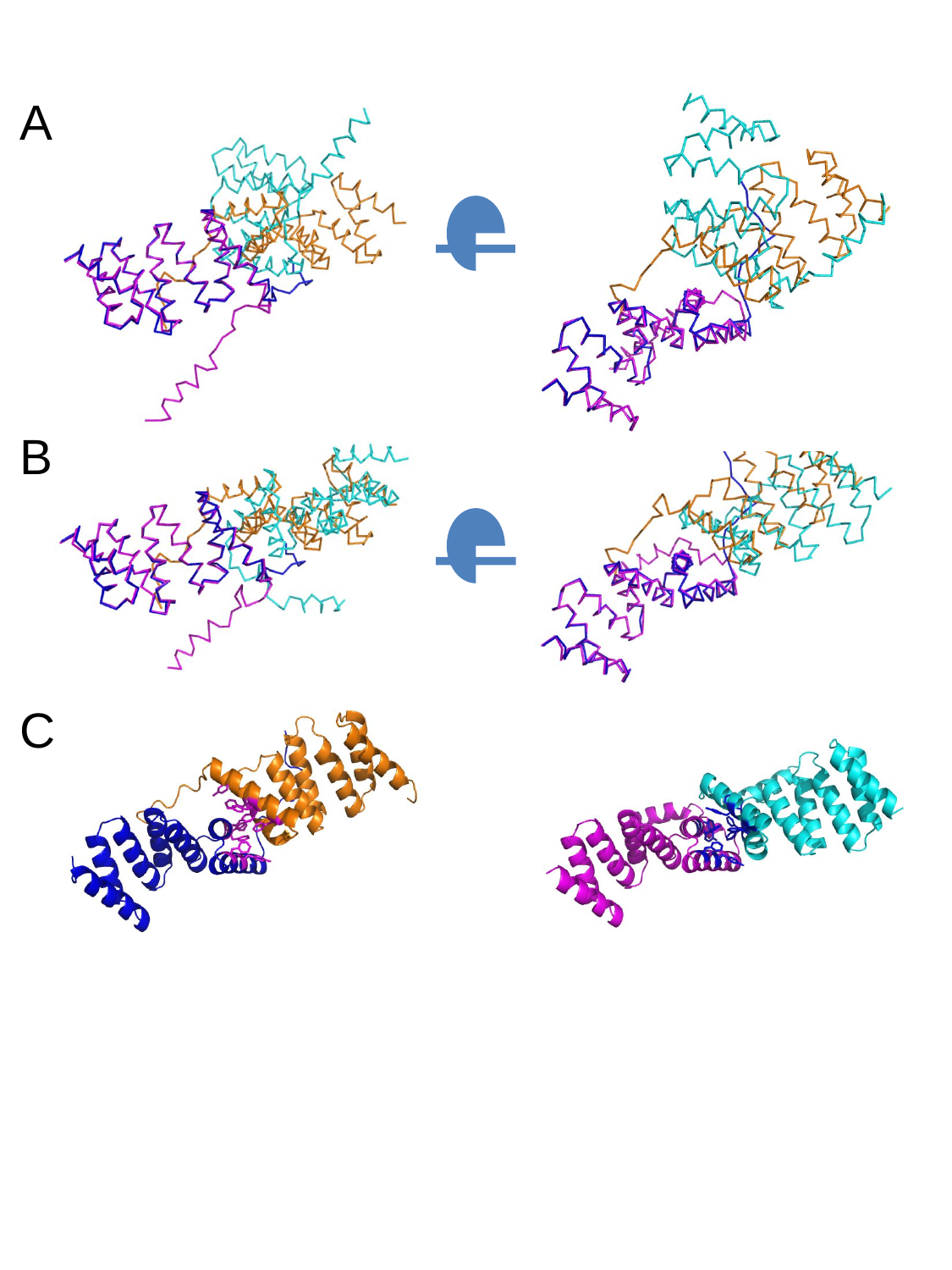

90°
A
B
90°
C

Supplement: Additional file 3 — Comparison of IpgC10-155 Dimers to Head-to-Head Dimer Assemblies Present in IpgC1-151 Crystals. Both the free (3GYZ) and IpaB peptide-bound (3GZ1) structures of IpgC1-151 were examined for potential "head-to-head" dimerization contacts similar to those observed in the IpgC10-155 structure presented in Figure 1. (A) Two orthogonal views of a putative dimer (magenta and cyan) from 3GZ1 superimposed with a prototypic dimer of IpgC10-155(blue and orange). This dimer is generated by applying the crystallographic symmetry operator x,-y+1,-z, and buries 666.9 Å2 of surface area upon formation. (B) Two orthogonal views of a putative dimer from 3GYZ superimposed with the IpgC10-155 dimer; all chains are colored as in panel A. This dimer is generated by applying the crystallographic symmetry operator x-y,-y,-z+2/3, and buries 950.4 Å2 of surface area upon formation. (C) Two identical views of the IpgC10-155 dimer (left) and the symmetry-generated dimer from 3GYZ shown in panel B (right). The set of aromatic residues described in Figure 3 are colored magenta and blue in the left and right panels, respectively. [file 1472-6807-10-21-S3.PPT]

## Slide 1
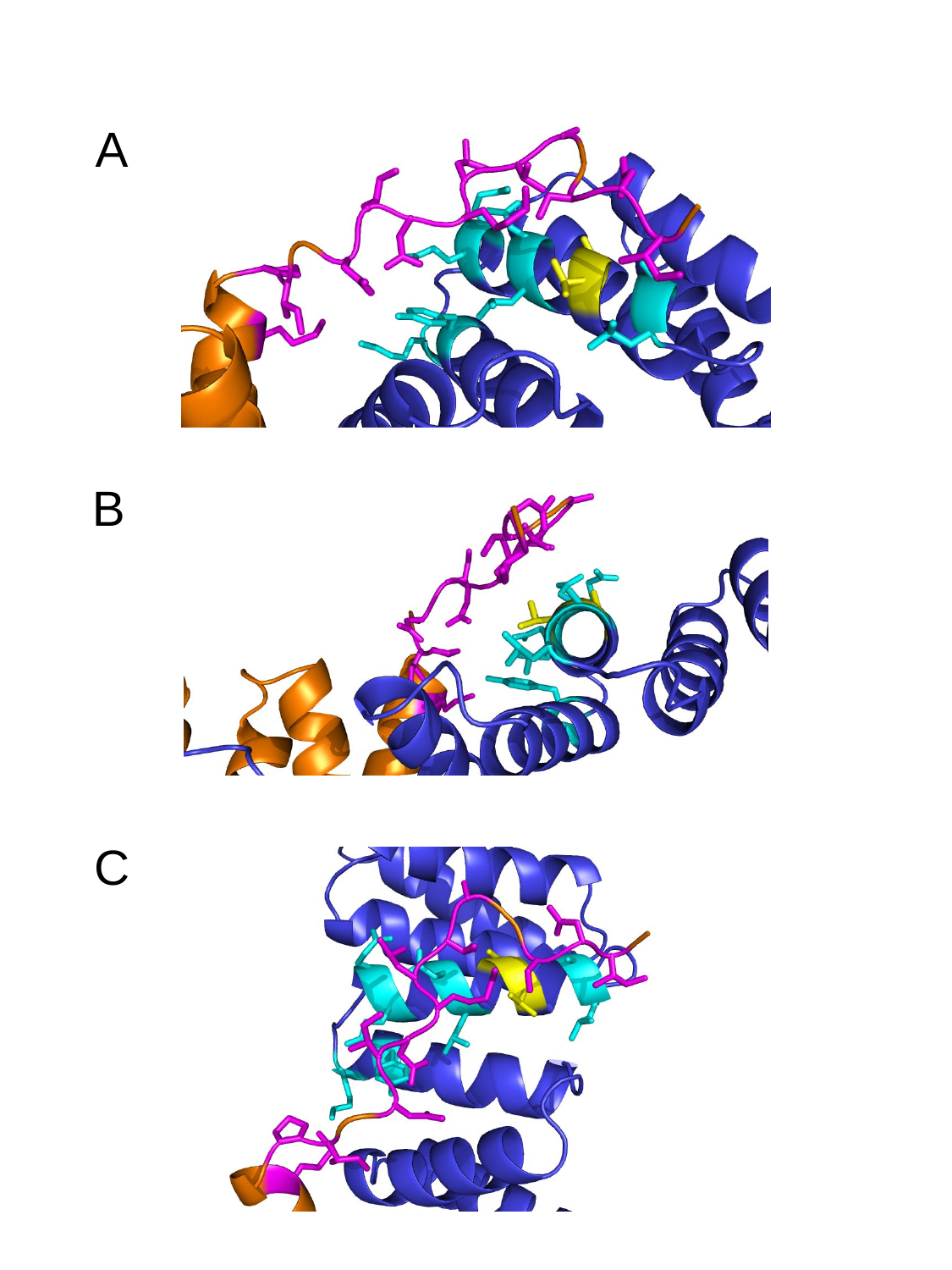

A
B
C

Supplement: Additional file 4 — Magnified View of the Interface between the Amino-terminal Region and Helix α5 in an IpgC10-155 Dimer. The individual monomers that comprise the IpgC10-155 dimer are depicted as blue and orange cartoons, while the residues contributing to buried surface area (ball-and-stick) in this region are colored in magenta and cyan, respectively. Residues Ala94 and Val95, which are critical for dimerization in IpgC1-151 [8], are colored yellow for reference. (A) Rendering of the IpgC10-155 dimer from a viewing plane opposite that of Figure 1B, C. (B) Rotated view of panel A such that the axis of helix α5 is orthogonal to the plane of the page. Note the packing of the amino-terminal residues (magenta) against the sidechains of α5 (cyan and yellow). (C) Similar to panel B, but the viewing plane has been rotated 90° with respect to the page. The zigzag nature of the packing between the magenta-colored amino-terminus and the opposing monomer is evident in this image. [file 1472-6807-10-21-S4.PPT]

## Slide 1
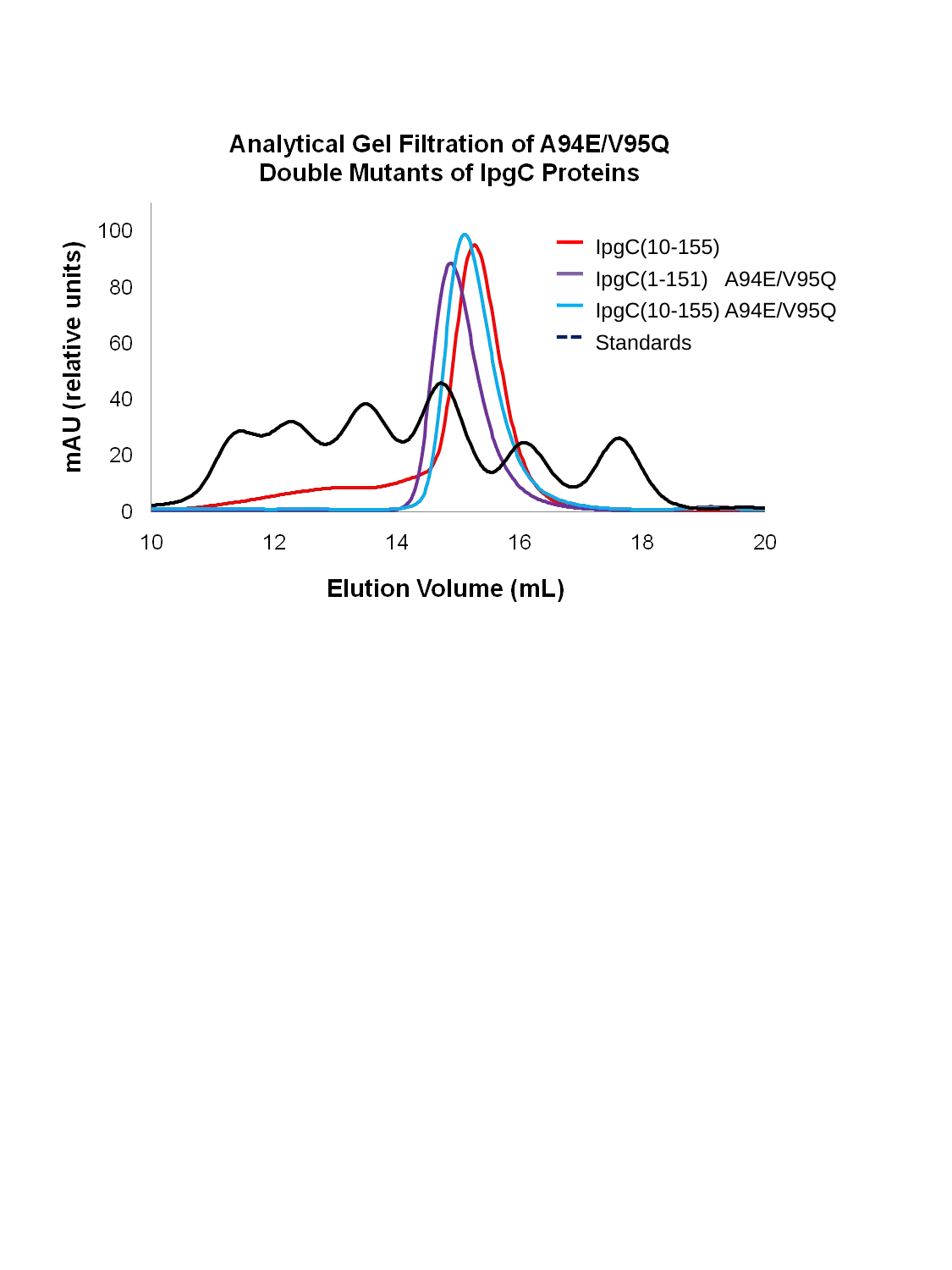

IpgC(10-155)
IpgC(1-151) A94E/V95Q
IpgC(10-155) A94E/V95Q
Standards

Supplement: Additional file 5 — IpgC Double Mutants Exist as Dimers in Solution. Description: Samples of purified IpgC (5 mg/mL) proteins were injected onto an analytical gel-filtration column and the elution profiles were compared to a series of known standards to derive an estimation of protein molecular weight (see Additional File 6, Figure S6). The sample identities are IpgC10-155 (red), IpgC10-155 Ala94Glu/Val95Gln (cyan), and IpgC1-151 Ala94Glu/Val95Gln (purple). The standard mixture is shown as a black dashed line. Aside from the standard injection, all curves were normalized to a maximum peak height of 100 mAU for clarity. [file 1472-6807-10-21-S5.PPT]

## Slide 1
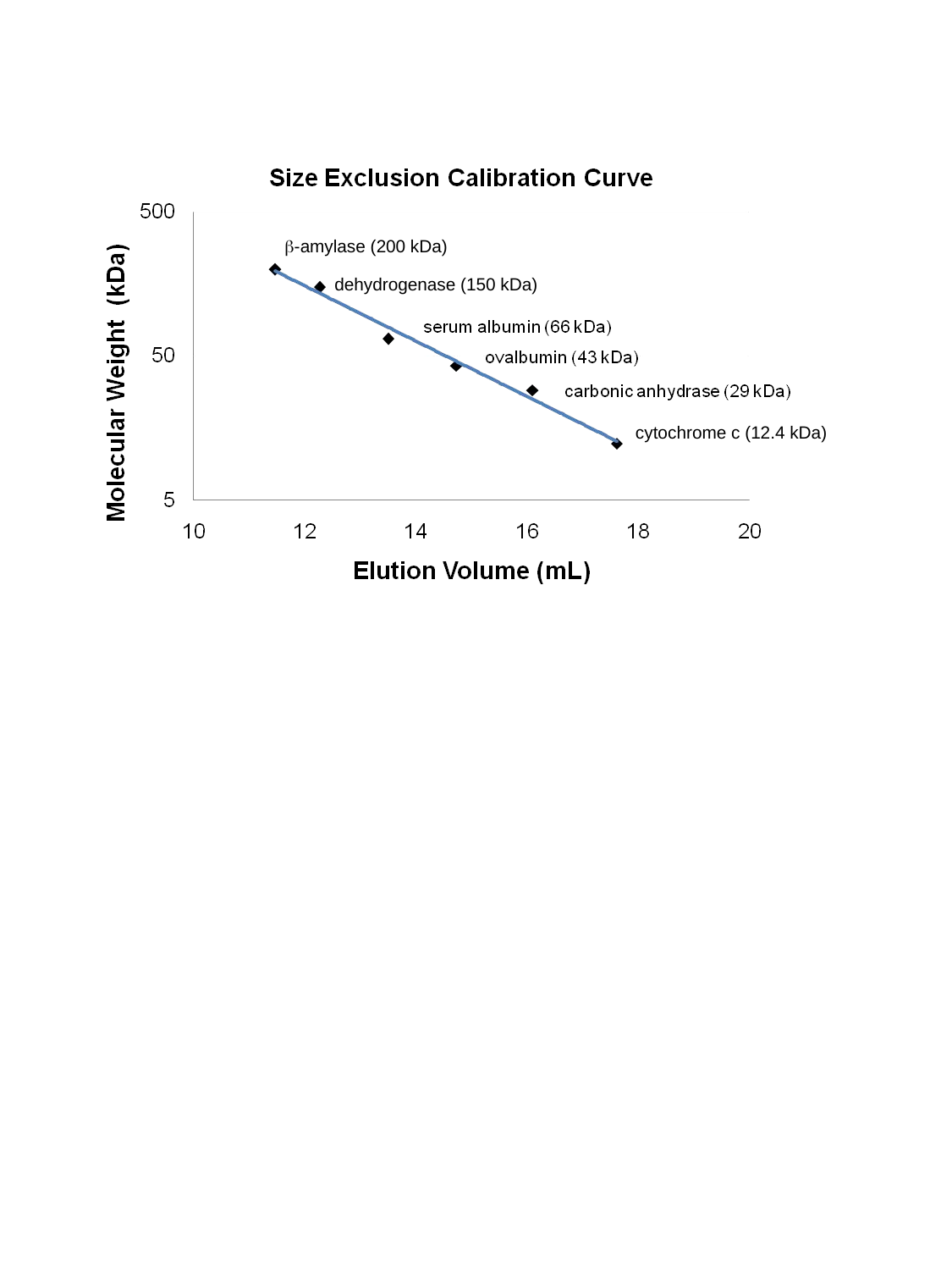

β-amylase (200 kDa)
dehydrogenase (150 kDa)
cytochrome c (12.4 kDa)

Supplement: Additional file 6 — Calibration of the Analytical Size Exclusion Chromatography Column. Size exclusion standard curve where observed molecular weight is plotted as a function of elution volume. Calibration points (black diamonds) correspond to the following standard proteins: β-amylase (200 kDa), dehydrogenase (150 kDa), serum albumin (66 kDa), ovalbumin (43 kDa), carbonic anhydrase (29 kDa) and cytochrome c (12.4 kDa). Estimations of molecular weight were determined using the relationship MW = 31.195×e(-0.443×(E.V.)), where the molecular weight (MW) is given in kDa and the elution volume (E.V.) is given in mL; the correlation coefficient for this relationship (R2) is 0.987 (blue line). [file 1472-6807-10-21-S6.PPT]
